# The Journey with paediatric cancer: reflections on its impact on patients and their families

**DOI:** 10.11604/pamj.2026.53.174.48283

**Published:** 2026-04-27

**Authors:** Neshika Munshi, Moeti Kgware, Raisuyah Bhagwan

**Affiliations:** 1 Department of Community Health Studies, Faculty of Child and Youth Care Programme, Durban University of Technology, Durban, KwaZulu-Natal, South Africa,; 2 Department of Community Health Studies, Faculty of Environmental Health, Durban University of Technology, Durban, KwaZulu-Natal, South Africa

**Keywords:** Caregivers, psychosocial impact, emotional distress, family burden, paediatric cancer, qualitative study

## Abstract

**Introduction:**

the experience of paediatric cancer presents intense emotional and psychological challenges for both children and their families. This study explores the lived experiences of parents caring for children with cancer at a public hospital in KwaZulu-Natal, South Africa, highlighting the multidimensional impact of the disease.

**Methods:**

a qualitative case study design was employed. Fifteen semi-structured interviews were conducted with parents of paediatric cancer patients at a tertiary public hospital in KwaZulu-Natal, South Africa. Data were transcribed verbatim and analysed manually using inductive thematic analysis.

**Results:**

two major themes emerged: the multidimensional effects of cancer on the child, and the multifaceted impact on the family unit. Children exhibited signs of emotional despair, fear of death, academic disruption, social isolation, and distress related to medical procedures. Parents reported significant psychological trauma, financial hardship, disruption of daily life and career, and strained marital and family relationships. Lack of information about the disease also compounded stress among caregivers.

**Conclusion:**

paediatric cancer deeply affects not only the physical health of children but also their emotional well-being and family stability. The findings underscore the urgent need for integrated psychosocial support, including mental health services and caregiver education, within paediatric oncology care settings.

## Introduction

Cancer remains one of the foremost causes of mortality worldwide, and approximately 400,000 new cases of childhood and adolescent cancer are reported each year [1]. Despite advancements in treatment that have improved survival outcomes, the emotional and psychosocial toll of childhood cancer remains profound. The diagnosis alone initiates a cascade of stress, fear, and uncertainty that affects both the young patients and their families [2]. For children diagnosed with cancer, the journey is emotionally taxing. They experience psychological distress, including fear of death, anxiety about medical procedures, and sadness linked to the disruption of their daily lives [3]. Many children face painful treatments, experience changes in physical appearance, and endure frequent hospitalizations that separate them from familiar routines, peers, and environments. This loss of normalcy contributes to a deep emotional vulnerability [4]. Feelings of despair, hopelessness, and emotional withdrawal become common, exacerbated by the invasive nature of treatment protocols. The visible side effects of cancer treatment, such as hair loss and physical weakness, often contribute to social stigma and alienation [5]. Children undergoing treatment may struggle with self-image and identity, leading to lowered self-esteem and difficulty reengaging with peers. As a result, many children withdraw socially, preferring isolation to the possibility of rejection or misunderstanding [6]. Even when physical health improves, reintegration into school and social circles remains challenging due to these psychological scars. Academically, the implications are just as severe. Prolonged treatment regimens and immunosuppression frequently necessitate long absences from school [7].

This academic disruption can result in significant learning gaps, cognitive delays, and difficulty keeping pace with peers. Even when children attend school, fatigue and “chemo brain” a common term for treatment-induced cognitive dysfunction, affect their ability to focus and retain information [8]. Falling behind academically often intensifies feelings of inadequacy and emotional distress, as education is a key domain in children´s lives that fosters routine, development, and social interaction [9]. Anxiety and depression are two of the most prevalent psychological responses among paediatric cancer patients [10]. The chronic stress of managing a life-threatening illness, combined with the emotional toll of constant monitoring, tests, and uncertain outcomes, manifests as deep-seated emotional instability. Many children exhibit signs of post-traumatic stress disorder, including flashbacks, hypervigilance, and fear associated with medical environments [11]. These individual challenges ripple outward to affect the entire family. Parents, in particular, face significant emotional strain [12]. The initial shock of the diagnosis often evolves into chronic anxiety and psychological fatigue as they grapple with caregiving responsibilities, complex medical information, and the prospect of losing their child [13]. Feelings of guilt, helplessness, and despair are common, especially when they must balance the needs of the sick child with other familial and professional obligations. In many cases, parents must alter their employment status to accommodate hospital visits and caregiving duties [14]. This often results in financial strain, particularly for single-income households or those without adequate health insurance. Costs associated with travel, accommodation near hospitals, medications, and time off work quickly accumulate, pushing families into economic vulnerability. These financial challenges compound the emotional burden, leading to heightened stress levels and, in some cases, breakdowns in familial relationships [15].

Siblings of paediatric cancer patients are also significantly affected. They may feel neglected due to the shift in parental attention, leading to emotional and behavioral issues [16]. The family´s daily routines are disrupted, and household dynamics are altered, often permanently. Parents frequently report feeling torn between caring for the sick child and maintaining a sense of normalcy for the rest of the family [17]. Cumulatively, these challenges underscore the far-reaching impact of paediatric cancer, which extends well beyond the physical implications of the disease. In addition to the medical battle, families face an emotional and psychological struggle that requires comprehensive support [18]. A holistic approach to paediatric oncology care is an essential one that integrates mental health services, academic support, financial counselling, and social reintegration programs for both the patient and their family members [19]. This study, rooted in the South African context, contributes critical insight into the mental health and psychosocial sequelae of paediatric cancer [20]. While global literature has highlighted similar issues, the specific cultural, social, and economic conditions faced by South African families call for targeted interventions and policy responses. Understanding these complex dynamics is vital for the development of sensitive, inclusive, and sustainable care strategies that support healing not just medically but emotionally and socially as well [21].

## Methods

**Study design and setting:** this study adopted a qualitative case study design to explore parents´ lived experiences of caring for a child diagnosed with cancer and to understand how paediatric cancer affected both the child and the family unit. The study was conducted at Inkosi Albert Luthuli Central Hospital, a tertiary public referral hospital in KwaZulu-Natal, South Africa, which provides specialist paediatric haematology-oncology services to children from diverse urban, peri-urban, and rural communities. Many families accessing care at this facility depend on public health services and may experience socio-economic constraints, including transport difficulties, employment insecurity, and limited access to psychosocial support, all of which shaped the context within which caregiving experiences were narrated. Data collection took place after ethics approval and institutional permissions had been secured.

**Study population and sampling:** a purposive sampling strategy was used to recruit 15 parents of paediatric cancer patients receiving treatment at the study site. The sample comprised 13 mothers and 2 fathers between 27 and 52 years of age. Participants were selected because they were primary caregivers of children diagnosed with cancer and were therefore able to provide detailed accounts of the psychosocial and family impact of the illness. Recruitment was facilitated through the Head of the Paediatric Haematology-Oncology Ward, who assisted in identifying potentially eligible participants. Thereafter, the researcher approached parents directly, explained the purpose of the study, provided the information letter, and invited voluntary participation. Parents were given an opportunity to ask questions before written informed consent was obtained. Inclusion criteria were parents or primary caregivers of a child diagnosed with cancer and receiving treatment at Inkosi Albert Luthuli Central Hospital, willingness to participate in an interview, and ability to provide informed consent. Sampling continued until sufficient depth and recurrence of experiences were reached across interviews.

**Data collection procedure:** data were collected through face-to-face semi-structured interviews conducted by the researcher using a common interview guide to ensure consistency while allowing participants to elaborate on their experiences. Interviews explored parental perceptions of the child´s emotional and physical suffering, family disruption, caregiving burden, financial strain, and coping experiences. Each interview lasted approximately 45 to 60 minutes, was audio-recorded with permission, and was conducted in a private space within the hospital to protect confidentiality. Audio recordings were transcribed verbatim soon after each interview. Field notes were written after interviews to document contextual observations, non-verbal cues, and early analytic reflections that later informed interpretation.

**Researcher reflexivity:** as the researcher was the primary instrument of data collection and analysis, reflexive awareness was maintained throughout the study. The researcher remained attentive to how personal assumptions, professional orientation, and emotional responses to parents´ narratives could shape data collection and interpretation. A reflexive journal was kept during the research process to document preconceptions, decisions made during interviewing and analysis, and reflections on how the researcher´s presence may have influenced participants´ responses. There was no prior personal relationship between the researcher and participants beyond the research encounter. Efforts were made to adopt a neutral, empathetic, and non-judgemental stance during interviews so that participants could describe their experiences in their own terms.

**Data analysis:** data were analysed using inductive thematic analysis. Analysis began immediately after transcription and followed a systematic multi-step process. First, each transcript was read and re-read several times to achieve immersion in the data and to gain an overall sense of participants´ experiences. Second, initial codes were generated manually by working line by line through each transcript and identifying meaningful units of text relevant to the research objective. Third, similar codes were grouped together and compared across transcripts to identify patterns of shared meaning. Fourth, these code clusters were organised into preliminary themes and subthemes that captured recurring dimensions of the participants´ experiences. Fifth, the preliminary themes were reviewed against the coded extracts and the full data set to ensure internal coherence and distinction between themes. Finally, themes were refined, named, and defined to reflect the central organising concepts emerging from the interviews. Manual coding was used instead of qualitative software to allow close engagement with participants´ narratives; however, rigor was maintained through a structured coding framework, dated coding sheets, code lists, theme matrices, and an audit trail documenting analytic decisions from initial coding to final theme development.

**Trustworthiness:** several strategies were used to enhance the trustworthiness of the findings. Credibility was strengthened through prolonged engagement with the interview data, repeated transcript review, and the use of verbatim quotations in the full manuscript to ground interpretations in participants´ accounts. Dependability was supported by maintaining a clear audit trail of methodological and analytic decisions, including coding records, theme development notes, and reflexive memos. Confirmability was enhanced through reflexive journaling and supervisory review of the coding structure and thematic interpretations to minimise the influence of individual bias. Transferability was addressed by providing contextual detail about the study setting, participant characteristics, and the caregiving environment so that readers may judge the relevance of the findings to similar settings. Member checking of full transcripts or final themes was not undertaken because of the clinical and emotional burden experienced by participants during the treatment period; this is acknowledged as a limitation. However, during interviews, the researcher used probing and paraphrasing to confirm the meaning of participants´ responses in real time. Although formal triangulation across multiple data sources was not conducted, emerging interpretations and theme structure were discussed with the supervisory team as a form of peer debriefing to strengthen analytic rigor.

**Ethical considerations:** ethical approval was obtained from the Institutional Research Ethics Committee of the Durban University of Technology, South Africa (Reference number: IREC 043/18). Additional permission was obtained from the relevant health authorities and hospital management. Written informed consent was obtained from all participants before data collection commenced. Participation was voluntary, confidentiality and anonymity were maintained, and participants were informed of their right to withdraw from the study at any stage without penalty. All recordings and transcripts were stored securely and accessed only for research purposes.

## Results

Two key themes emerged from the data: i) the multidimensional effects of cancer on the paediatric patient and ii) the multifaceted impact of paediatric cancer on the family unit [22].

**Theme 1: the multidimensional effects of cancer on the paediatric patient:** the first theme highlights the diverse and interconnected effects of cancer on paediatric patients, extending beyond physical illness to include emotional, psychological, social, academic, and developmental dimensions. Seven subthemes were identified, reflecting the breadth of this impact [23].

**Subtheme 1: emotional despair and distress:** parents described the emotional toll of cancer on their children as profound and distressing. Children who were previously active and cheerful became withdrawn, anxious, and emotionally unsettled. One parent observed that her child became panicked before hospital visits, while another noted a marked disengagement from school and social activities. These emotional changes reflected a significant shift in behaviour and mood, illustrating how the illness disrupted children´s sense of normalcy and emotional stability. Parents consistently described their children as experiencing sadness, fear, and emotional vulnerability throughout the treatment process.

**Subtheme 2: fear of death:** children living with cancer were described by parents as experiencing a persistent and overwhelming fear of death. Parents reported that their children became highly sensitive to physical symptoms, often interpreting minor changes in their bodies as signs of worsening illness. Some children expressed anxiety about dying, while others avoided conversations related to death altogether [24]. This fear appeared to shape their emotional responses, contributing to heightened distress, uncertainty, and ongoing anxiety about their health and future.

**Subtheme 3: the impact on school life:** cancer significantly disrupted children´s academic trajectories. Parents reported prolonged absences from school, missed academic progress, and concerns about reintegration [25]. Visible treatment-related changes, such as hair loss or physical weakness, contributed to fears of stigma and bullying. These challenges affected children´s confidence, participation, and sense of achievement within the school environment.

**Subtheme 4: social isolation and withdrawal:** social isolation emerged as a major challenge. Due to hospitalisation and infection control measures, children experienced limited interaction with peers and, in some cases, loss of friendships. Parents observed increasing loneliness and emotional withdrawal, which affected children´s sense of belonging and overall emotional well-being.

**Subtheme 5: fear of medical procedures:** medical procedures, particularly those involving needles, were identified as a significant source of anxiety. Parents described intense distress when children encountered clinical environments, including fear of injections and resistance to treatment procedures [26].

**Subtheme 6: fear of the unknown and recurrence:** uncertainty regarding treatment outcomes and the possibility of recurrence contributed to ongoing anxiety [27]. Parents reported that children often felt confused and fearful about their future, expressing concern about whether they would recover or become ill again.

**Subtheme 7: physiological effects of cancer and treatment:** the physical effects of cancer and its treatment were described as severe and debilitating. Children experienced fatigue, vomiting, weakened immunity, and visible changes such as hair loss. Parents frequently reported that their children were persistently tired or unwell, and these physical challenges further contributed to emotional distress.

**Theme 2: multifaceted impact of pediatric cancer on the family unit:** the second theme reflects the wide-ranging impact of paediatric cancer on the family unit. Parents described how the child´s illness extended beyond the individual patient to affect emotional well-being, financial stability, family functioning, relationships, and future life plans. Seven interrelated subthemes were identified, reflecting the cumulative burden experienced by caregivers and households.

**Theme 2: multifaceted impact of paediatric cancer on the family unit**

**Subtheme 1: mental health distress encountered by parents:** parents of children with cancer frequently endured significant psychological distress. Constant exposure to their child´s suffering triggered emotions of fear, trauma, anxiety, and helplessness. One mother, who had previously lost a child to liver cancer, described an overwhelming fear of experiencing such a loss again. Another parent highlighted the emotional strain associated with fluctuating blood count levels and repeated treatment setbacks. In addition to caring for the ill child, many parents reported distress related to leaving their other children in the care of extended family members, often resulting in guilt and emotional conflict [28]. These experiences reflect the intense and layered emotional burden associated with caregiving.

**Subtheme 2: financial difficulties:** the financial strain associated with childhood cancer was substantial. Many parents were forced to leave employment or reduce working hours to provide full-time care, resulting in a significant decline in household income. One mother recounted leaving her job to remain with her hospitalised child, leading to financial hardship and loss of independence. Even where some financial support existed, the cumulative costs of treatment, travel, and accommodation placed considerable pressure on families. In some cases, savings were depleted and income-generating activities ceased entirely. These financial challenges extended beyond the immediate caregiving context, affecting the well-being and opportunities of other family members [29].

**Subtheme 3: disruption of normal family life:** childhood cancer disrupted the structure and functioning of family life. Parents described changes in daily routines, caregiving responsibilities, and living arrangements, including relocation closer to treatment facilities. These adjustments often resulted in job losses, strained relationships, and reduced attention to other children. Some parents reported behavioural changes in siblings, linked to reduced parental presence and emotional support. The cumulative effect of these disruptions placed families under sustained pressure, affecting both emotional connection and overall stability [30].

**Subtheme 4: instability within the marital relationship:** marital relationships were significantly affected by the demands of caregiving. Parents reported emotional distancing, communication challenges, and reduced intimacy. In some cases, prolonged stress and unequal caregiving responsibilities contributed to relationship breakdown. Participants described how the focus on the ill child limited opportunities for mutual support, placing additional strain on the partnership and altering family dynamics.

**Subtheme 5: pain and anguish of mothers:** mothers, in particular, described profound emotional suffering characterised by grief, guilt, and exhaustion. One mother explained how her world felt shattered following her child´s diagnosis, while others expressed ongoing emotional distress linked to uncertainty and fear of loss. Many mothers carried primary caregiving responsibilities, managing both medical demands and emotional support. This dual burden contributed to sustained psychological strain and reshaped their personal and maternal identities [31].

**Subtheme 6: disruption of academic and career trajectories:** the diagnosis of childhood cancer significantly disrupted parents´ educational and professional pathways. Some parents were forced to withdraw from academic programmes or postpone career advancement to focus on caregiving. One participant described abandoning her legal training after her child´s diagnosis, resulting in financial and personal setbacks. These disruptions often extended beyond the treatment period, affecting long-term socio-economic stability.

**Subtheme 7: lack of knowledge regarding the child´s cancer diagnosis:** a recurring challenge for parents was limited understanding of the child´s diagnosis and treatment process. Many reported difficulty interpreting medical terminology and treatment plans, particularly at the initial stage of diagnosis. Some relied on external sources, such as the internet, to make sense of the condition [32]. This lack of clarity contributed to increased anxiety and uncertainty, affecting parents´ confidence in decision-making and caregiving.

## Discussion

This study explored the multidimensional impact of paediatric cancer on children and their families within a South African context. The findings highlight the interconnected emotional, psychological, social, and financial burdens associated with the illness, reinforcing existing literature while contributing context-specific insights from a public-sector healthcare setting [33]. Children in this study experienced profound emotional distress, including fear of death, anxiety related to treatment procedures, and uncertainty about health outcomes. These findings align with previous research describing how childhood cancer disrupts a child´s sense of safety, identity, and emotional stability [34]. The reported social withdrawal and school absenteeism further demonstrate how the illness interferes with key developmental domains, including education and peer relationships, thereby affecting long-term psychosocial adjustment. Fear of medical procedures and uncertainty about recurrence emerged as persistent stressors. These findings are consistent with existing evidence that highlights the long-term psychological impact of invasive treatments and ongoing uncertainty, even after the completion of therapy [35]. The continuation of such fears underscores the importance of sustained psychological support for paediatric patients throughout and beyond the treatment trajectory. The physiological effects of cancer and its treatment, including fatigue, vomiting, and weakened immunity, were found to compound emotional distress and limit children´s participation in daily activities such as schooling. Visible physical changes, particularly hair loss, further contributed to stigma and social isolation, reinforcing the interconnected nature of physical and psychosocial challenges.

The study also identified significant emotional distress among parents, particularly mothers, who reported feelings of fear, guilt, helplessness, and exhaustion. These findings are consistent with prior research indicating that caregiving in the context of childhood cancer is associated with long-term psychological strain [36]. The emotional burden was further intensified in cases where parents had previous experiences of child loss, highlighting the cumulative nature of trauma. Financial hardship emerged as a critical challenge, with many families experiencing reduced income alongside increased medical and caregiving expenses. These findings reflect broader evidence on the economic burden of childhood illness, demonstrating how financial strain can extend beyond the patient to affect siblings and overall household stability [37]. Strain within marital relationships was also evident, with participants describing emotional distancing, communication challenges, and, in some cases, relationship breakdown. These findings support existing literature indicating that chronic illness in children can disrupt family dynamics and weaken relational cohesion. In addition, disruptions to parents´ educational and professional trajectories further illustrate the long-term socio-economic consequences of caregiving [38].

A notable finding was the limited understanding of cancer diagnoses among parents, despite interactions with healthcare providers. This gap in knowledge contributed to increased anxiety and uncertainty, highlighting the need for improved communication strategies. Clear, accessible, and empathetic information delivery is essential to support informed decision-making and enhance caregiver confidence [39]. Overall, the findings emphasise the need for a holistic and multidisciplinary approach to paediatric oncology care. Interventions should extend beyond clinical treatment to include psychological support, social work services, financial counselling, academic reintegration programmes, and improved caregiver education. Addressing these interconnected needs is essential to improving both patient and family outcomes. The study should be interpreted in light of certain methodological considerations. Although the qualitative design enabled an in-depth understanding of parental experiences within a specialised public hospital context, the findings are based on a purposively selected sample from a single site and are therefore not intended for statistical generalisation. In addition, formal member checking of transcripts and themes was not undertaken due to the clinical and emotional demands placed on participants during treatment [40]. However, methodological rigor was strengthened through systematic thematic analysis, reflexive journaling, maintenance of an audit trail, and supervisory peer debriefing during the analytic process.

## Conclusion

This study examined how paediatric cancer affects children and their families within a public-sector tertiary hospital context in KwaZulu-Natal, South Africa. The findings demonstrate that the burden of childhood cancer extends beyond physical illness to include emotional suffering, fear, educational disruption, social withdrawal, caregiver distress, financial strain, and disruption of family relationships and future plans. By foregrounding parents´ accounts, the study shows that paediatric oncology care must be understood as both a clinical and psychosocial response to illness. The findings support the need for integrated family-centred interventions that include psychological support, caregiver education, social assistance, and clear communication from healthcare providers. These contextually grounded insights contribute to a fuller understanding of paediatric cancer care in resource-constrained settings and may assist oncology teams in developing more responsive and holistic support systems for children and their families.

### What is known about this topic

- Childhood cancer causes significant emotional and psychological distress in both patients and their families;
- Clinical research has primarily focused on treatment outcomes and survival rates;
- Paediatric cancer disrupts children’s schooling, peer relationships, and normal developmental trajectories.

### What this study adds

- Provides in-depth qualitative evidence on the psychological toll and emotional trauma experienced by caregivers in a South African context;
- Highlights the disruption of marital stability, career goals, and daily family routines as core impacts of paediatric cancer;
- Emphasizes the urgent need for integrated psychosocial support services within paediatric oncology wards.
